# Inflammatory pseudotumor associated with HSV infection of rectal vascular endothelium in a patient with HIV: a case report and literature review

**DOI:** 10.1186/s12879-020-04960-5

**Published:** 2020-03-19

**Authors:** Shi Bai, Justin A. Maykel, Michelle X. Yang

**Affiliations:** 1 Department of Pathology, University of Massachusetts Memorial Health Care, 1 Innovation Drive, Worcester, MA 01605 USA; 2 Department of Surgery, University of Massachusetts Memorial Health Care, Worcester, MA 01605 USA

**Keywords:** HSV1, HSV2, HIV, Proctitis, Inflammatory pseudotumor

## Abstract

**Background:**

Herpes simplex virus (HSV) typically infects oral or anogenital squamous epithelium and causes blisters and ulcerations. Here we reported an unusual case of HSV induced exuberant rectal inflammatory pseudotumor with vascular endothelial involvement.

**Case presentations:**

A 52-year old man with HIV presented with abdominal pain, rectal drainage and constipation. Proctoscopy and CT scans revealed an 8 × 5 × 4 cm circumferential, mid-lower rectal mass that was concerning for malignancy. PET-CT showed mild to moderate FDG uptake of the rectal mass. Repeated biopsies showed exuberant lymphoplasmacytic inflammation with rich eosinophils and necrosis in the submucosa and scattered single or multi-nucleated viral inclusions in vascular endothelial cells that were positive for HSV by immunostains. There was no evidence of malignancy on histology or by immunostains. The patient started valacyclovir for three weeks and symptoms resolved after the antiviral therapy. Follow-up CT and sigmoidoscopy with biopsy revealed no rectal mass or drainable collection.

**Conclusions:**

HSV may present as proctitis with exuberant inflammatory response and mass-like lesion, and damages vascular endothelial cells in patients with HIV. The HSV-associated mass-like lesion can be effectively treated by 3-week valacyclovir.

## Background

Herpes simplex virus (HSV) is subtyped into HSV1 and HSV2 and is one of the most common infections in the world. According to the Centers for Disease Control and Prevention report, the prevalence of HSV1 and HSV2 was 47.8 and 11.9%, respectively, among the 14–49 year old population during 2015–2016 in the United States [1]. HSV1 is usually transmitted by oral-oral contact and presents as oral herpes, whereas HSV2 is typically sexually transmitted and presents as genital or anal herpes [2], although recent studies showed anogenital HSV1 infection is surprisingly increasing [3, 4]. HSV tends to infect the squamous epithelium at the genital and perianal areas and causes ulcer and blisters [2]. However, atypical clinical presentations such as mass or nodular lesions in genital and perianal areas, especially in immunocompromised patients have been reported [5, 6]. Here, we reported a case of rectal mass lesion as a result of HSV infection of the vascular endothelium in a 52-year-old patient with HIV. The identification of viral cytopathic changes in endothelial cells confirmed by immunostains was essential for the correct diagnosis and therefore effective antiviral treatment for this patient in lieu of surgical resection for suspected rectal malignancy.

## Case presentations

A 52-year old male, who had a history of HIV currently on combined therapy of elvitegravir, cobicistat, emtricitabine and tenofovir DF and type 2 diabetes, initially presented at an outside hospital with abdominal pain, constipation with occasional blood in the stools and episodes of fecal incontinence for the past 3 months. He also had unintentional weight loss of approximately 10 pounds over the past 3 months. The outside hospital computed tomography (CT) scans revealed circumferential rectal wall thickening with enhancement and 9 cm air-fluid collection along the mid-lower rectum, which was initially suspicious of an intramural abscess. He was subsequently put on empiric antibiotics including levofloxacin plus metronidazole and later amoxicillin/clavulanate. However, his rectal symptoms persisted and rigid proctoscopy was performed to find a perforated necrotic rectal tumor that was clinically suspicious of malignancy. The outside biopsies showed colonic mucosa and submucosa with ulceration, necrosis, hemorrhages and dense lymphoplasmacytic chronic inflammation. There was no evidence of dysplasia or malignancy. Subsequent magnetic resonance imaging (MRI) revealed 8 × 5 × 4 cm necrotic rectal mass with enlarged mesorectal lymph nodes. CT-guided fine-needle aspiration was performed for the rectal wall mass and was negative for malignancy. Core biopsies were repeated and failed to demonstrate malignancy. With persisting symptoms, he was diverted with a sigmoid colostomy.

The patient was then referred to our institution for further management. His CEA was normal (< 0.5 ng/ml). Lymphogranuloma venereum and Chlamydia antibody tests were unremarkable. PET-CT showed mild to moderate FDG uptake of the rectal mass and mesorectal lymph nodes. A repeated biopsy was performed and showed similar inflammatory infiltrates and scattered viral cytopathic changes in the submucosal stromal cells. Extensive immunohistochemistry (IHC) studies were performed and revealed no evidence of lymphoma or carcinoma. However, HSV1 and HSV2 immunostains were positive in submucosal vascular endothelial cells, consistent with herpes simplex proctitis associated with an inflammatory pseudotumor. The patient therefore started oral valacyclovir 1 g twice a day for three weeks and rectal symptoms revolved after the treatment. The patient reported 20 lbs. weight gain after the antiviral therapy. Recent follow-up CT scans showed complete resolution of the rectal mass and no further drainable fluid collection. Post treatment flexible sigmoidoscopy revealed normal appearing rectal mucosa and healing anal mucosa with negative viral immunostains and the patient has been scheduled for colostomy closure.

### Histological findings

**Fig. 1:**
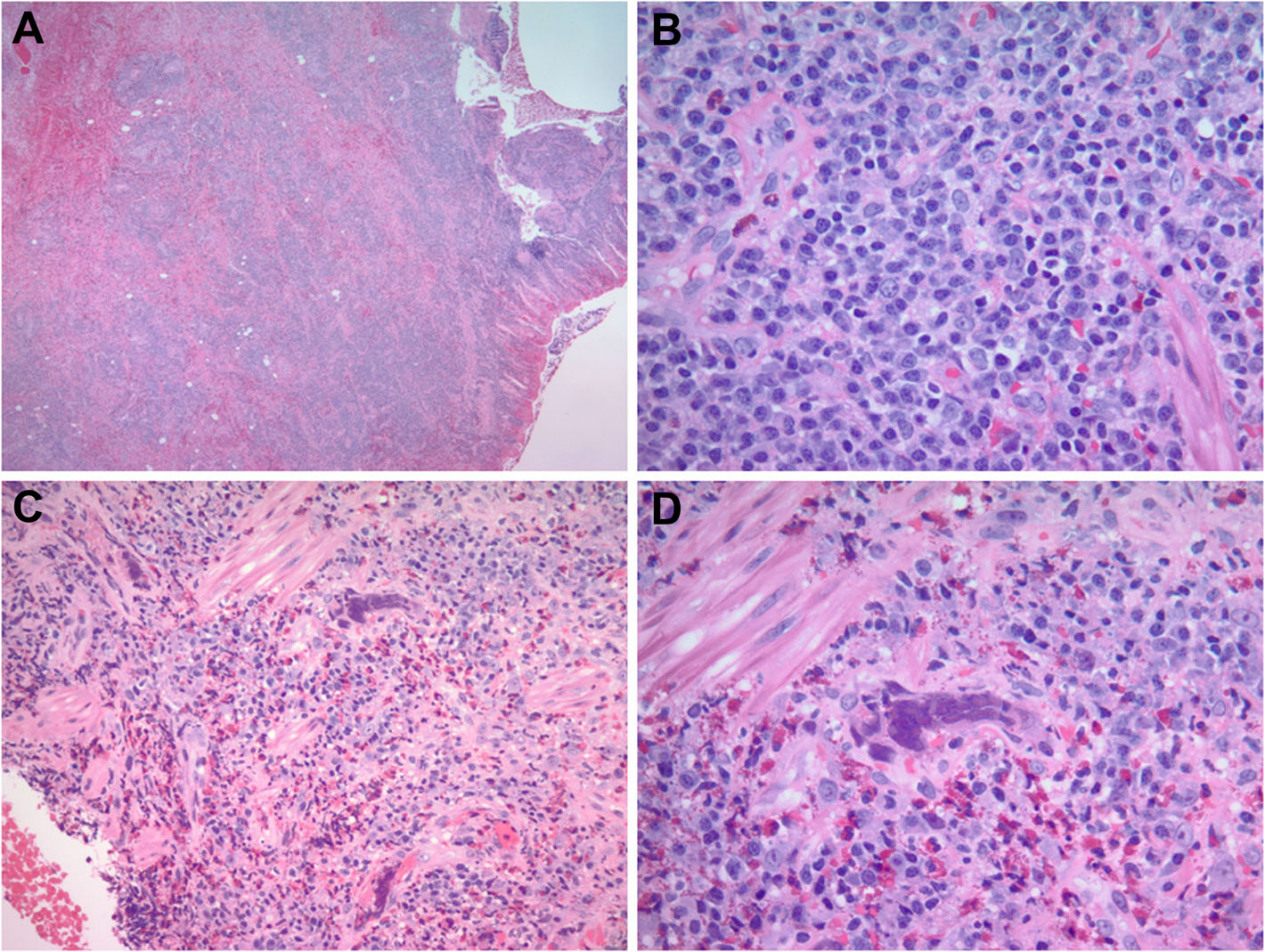
Hematoxylin and eosin stain (H&E) of rectal mass-like lesion in a patient with HIV. **a**. Ulcerated rectal mucosa with mass-like submucosal exuberant inflammatory infiltrate. **b**. Dense lymphoplasmacytic inflammation in submucosa. **c**. Rich eosinophilic infiltrate with single or multi-nucleated viral inclusions in endothelial cells. **d**. High power view of endothelial multi-nucleated inclusions. Original magnifications: **a**, 20X; **b**-**d**, 400X

**Fig. 2:**
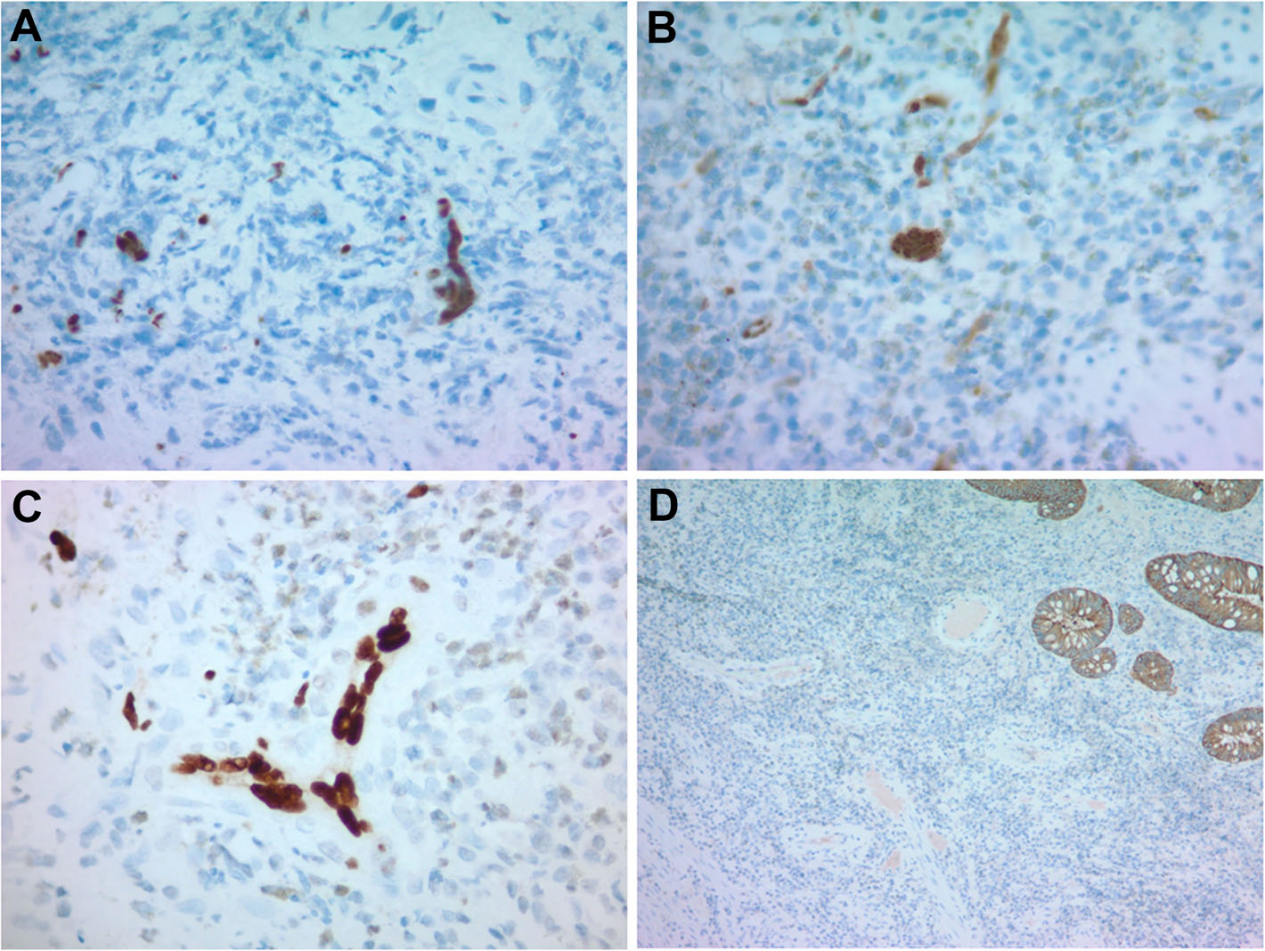
Immunohistochemistry of rectal mass-like lesion. **a**. HSV1, **b**. HSV2, and **c**. ERG positivity in vascular endothelial cells. **d**. Pan-keratin OSCAR highlighted surface non-neoplasia colonic epithelium. Original magnifications: **a**-**c**, 400X; **d**, 100X

The rectal mass biopsies showed similar histomorphology including colonic mucosa and submucosa with ulceration, necrosis, hemorrhages, acute and dense lymphoplasmacytic chronic inflammation, and eosinophilic infiltrate (Fig. 1a-b). Viral cytopathic changes were frequently seen in the submucosal vascular endothelial cells as single nuclear inclusion or multi-nucleated inclusions (Fig. 1c-d). Immunohistochemical stains demonstrated the viral inclusions were positive for HSV-1 (Fig. 2a) and HSV-2 (Fig. 2b). Viral infected single or multi-nucleated cells were positive for ERG by immunostain (Fig. 2c), supporting that the HSV infected cells were endothelial origin. Other viral immunostains such as adenovirus, *cytomegalovirus* (CMV), and human herpes virus 8 (HHV8) were negative. Epstein-Barr virus (EBV) in situ hybridization, spirochetes, acid fast bacilli (AFB) and PASD for fungus studies were also negative. CD138, kappa and lambda immunostains revealed polytypic plasma cells and no evidence of hematopoietic malignancy. Pan-cytokeratin immunostain supported negative for carcinoma (Fig. 2d). The overall morphology and immunostain results supported an inflammatory pseudotumor associated with HSV proctitis.

## Discussion and conclusions

HSV is one of the most prevalent infections in immunocompetent and immunocompromised patients. HSV1 and HSV2 typically affect anogenital squamous epithelium and present as genital or perianal ulcer and blisters, and proctitis mimicking inflammatory bowel disease in immunocompromised and homosexual man has been reported [7–10]. Anogenital HSV infections may less commonly cause tumor-like nodules or condylomatous lesion [5, 6]. HSV was recognized as the leading cause of proctitis in patients with HIV [11], and all reported tumor-like hypertrophic lesions caused by HSV including our case were patients with HIV [6, 12, 13]. Mass-like lesions caused by HSV infection can be a mimicker of malignancy clinically and have been reported in other anatomic sites such as nasal cavity [12], bronchial [14], and conjunctiva [15]. Interestingly, the first case of HSV1 proctitis mimicking rectal cancer in a patient with HIV has recently reported in English literature by Ayoade et al. [13], and biopsies revealed similar lymphoplasmacytic and eosinophilic infiltrate seen in our patient. However, in Ayoade’s case report, the viral nuclear inclusions were identified in colonic epithelial cells that were positive for HSV1 by immunostain [13]. In contrast, our case showed no HSV cytopathic inclusions within the colonic epithelium, but single and multi-nucleated HSV inclusions were identified in the submucosal vascular endothelial cells, and were associated with submucosal hemorrhages, necrosis and perforation of the rectal wall. To our knowledge, this is the first case of inflammatory pseudotumor associated with HSV endothelial infection in English literature. Mechanistically, in vitro study has suggested that HSV1 can induce complement C3b receptor on a variety of cell types including epithelial, endothelial and fibroblastic cells to mediate HSV1 viral glycoprotein C, therefore infection of these cells [16]. Therefore, searching for viral cytopathic changes in the colonic epithelium, squamous cells if present, vascular endothelial cells and stromal cells was essential to rule out HSV infection.

In conclusion, HSV may rarely present as proctitis with exuberant inflammatory response and mass-like lesion, and damages vascular endothelial cells in patients with HIV. Comprehensive workup must be completed to avoid surgical resection for a presumed malignancy. These HSV-associated mass-like lesions can be effectively treated by a 3-week course of oral valacyclovir.

## Data Availability

Not applicable.

## Abbreviations

HIV: Human Immunodeficiency Virus
HSV: Herpes Simplex Virus
CT: Computed Tomography
CMV: *Cytomegalovirus*
ERG: ETS-Related Gene
HHV8: Human Herpes Virus 8
EBV: Epstein-Barr Virus (EBV)
AFB: Acid Fast Bacilli
PASD: Periodic Acid–Schiff with Diastase
